# Local Gene Transfer of OPG Prevents Joint Damage and Disease Progression in Collagen-Induced Arthritis

**DOI:** 10.1155/2013/718061

**Published:** 2013-10-03

**Authors:** Qingguo Zhang, Weiming Gong, Bin Ning, Lin Nie, Paul H. Wooley, Shang-You Yang

**Affiliations:** ^1^Orthopaedic Research Institute, Via Christi Wichita Hospitals, Wichita, KS 67214, USA; ^2^Jinan Central Hospital, Shandong University, Jinan 250013, China; ^3^Qilu Hospital of Shandong University, Jinan 250012, China; ^4^Department of Biological Sciences, Wichita State University, 1845 N Fairmount Street, Wichita, KS 67260, USA

## Abstract

This study examined the influence of osteoprotegerin (OPG) gene transfer on a murine collagen-induced arthritis model. A single periarticular injection of AAV-OPG or AAV-LacZ on the arthritic paw successfully incorporated the exogenous gene to the local tissue and resulted in marked transgene expression in the joint homogenate for at least three weeks. Clinical disease scores were significantly improved in OPG treated mice starting at 28-day post-treatment (*P* < 0.05). Histological assessment demonstrated that OPG gene transfer dramatically protected mice from erosive joint changes compared with LacZ controls (*P* < 0.05), although treatment appeared less effective on the local inflammatory progress. MicroCT data suggested significant protection against subchondral bone mineral density changes in OPG treated CIA mice. Interestingly, mRNA expressions of IFN-g and MMP3 were noticeably diminished following OPG gene transfer. Overall, gene transfer of OPG effectively inhibited the arthritis-associated periarticular bone erosion and preserved the architecture of arthritic joints, and the study provides evidence that the cartilage protection of the OPG gene therapy may be associated with the down-regulation of MMP3 expression.

## 1. Introduction

Rheumatoid arthritis (RA) is one of the most common forms of arthritis that affects more than two million Americans. It is characterized by chronic inflammation of the joint(s) with severe pain and swelling, impaired functions, and eventually joint distortion [1–3]. Bone destruction is a frequent and clinically serious event in patients with RA. Studies have shown that many proinflammatory cytokines and mediators such as macrophage colony-stimulating factor (M-CSF), the receptor activator of nuclear factor *κ*B ligand (RANKL), TNF, IL-1, and IL-17 play dominant roles in the pathogenesis of arthritis-associated bone loss [1, 3]. RANKL expression was documented at the sites of active bone erosions [4]. The main sources of RANKL are osteoblasts but RANKL is also expressed in synovial cells, activated T cells, mature B cells, and natural killer cells [5–8]. Expression of RANKL is up-regulated by parathyroid hormone, 1,25(OH)_2_D3, and several proinflammatory cytokines, including IL-1, IL-6, IL-17, and TNF-*α* [9–12]. RANKL functions both as a membrane anchored molecule and a soluble molecule. Both forms bind to RANK, the receptor of RANKL. RANKL and RANK are members of the TNF superfamily [13, 14]. RANK is expressed on osteoclast precursors and mature osteoclasts [15]. RANKL-RANK interaction is an essential factor during osteoclastogenesis and bone resorption [16]. Pettit et al. [17] reported that RANKL-deficient mice were protected from bone erosions in an experimental mouse arthritis, and similarly the RANKL-knockout mice developed severe osteopetrosis due to a complete lack of osteoclastogenesis [18]. RANKL also serves as survival factor and activates osteoclasts, suggesting its important role in the progression of rheumatoid arthritis.

 Osteoprotegerin (OPG) is a naturally existing decoy receptor that binds RANKL without physiological signal transduction. OPG was initially identified as a soluble factor that was capable of inhibiting osteoclastogenesis in vitro [19, 20]. It binds to RANKL and competes for RANKL with RANK, which blunts osteoclastogenesis and blocks its biological signal transduction. OPG-deficient mice exhibit severe osteoporosis [21]. The significance of RANKL-RANK-OPG signaling in regulating the immune system of bone continues to emerge. The balance of RANKL to OPG ratio regulates osteoclastogenesis and bone modeling. In the complex system of bone remodeling, the RANKL/OPG pathway is the coupling factor between bone resorption and formation [22]. Ultimately, the balance between RANKL and OPG determines the degree of proliferation and activity of the osteoclasts [14].

Our hypothesis is that restoration of the ratio of RANKL: OPG by administration of exogenous OPG may be a potential therapy to protect against bone loss in RA. Due to the short half-life of biological agents and the chronic nature of the disease, gene therapy provides an attractive alternative to deliver gene to arthritic site where therapeutic protein (OPG) could be locally produced and executed. In this study, we examined the feasibility and efficacy of the adeno-associated virus (AAV)—mediated OPG gene transfer in a mouse collagen induced arthritis (CIA) model. 

## 2. Materials and Methods

### 2.1. Animals

Female DBA/1 mice were obtained from Jackson Laboratory (Bar Harbor, ME) and housed in our pathogen-free barrier facility (12 hr light/12 hr dark cycle) and fed rodent chow. All animals were quarantined in the facility for two weeks prior to experimentation. The study was approved by the institutional Animal Investigation Committee (AIC).

### 2.2. Recombinant AAV Vectors

Generation and purification of the AAV-OPG-eGFP plasmid has been described previously [23]. The purified pAAV-CMV-OPG-IRES-EGFP was sent to the Gene Core Facility, University of North Carolina at Chapel Hill for rAAV-OPG-IRES-EGFP packaging and purification using a helper virus-free method [24]. The titer of the resulting rAAV-OPG-IRES-EGFP was ~1 × 10^9^/mL. AAV-LacZ vectors were purchased from the Gene Core Facility, University of North Carolina at Chapel Hill, with original titer of 1 × 10^11^/mL.

### 2.3. Induction and Assessment of CIA

The establishment of the CIA model was detailed previously [25]. Briefly, forty (40) female DBA/1 mice at 10–12 weeks of age were intradermally injected 100 *μ*g type II bovine collagen (kindly provided by Dr. Marie Griffiths, University of Utah) in Freund's complete adjuvant (FCA) at the base of the tail, followed by daily examination for the onset of the disease. Onset of CIA was determined upon observation of the first signs of definitive erythema and edema in the metatarsal or metacarpal regions of the paws. 100% of mice developed arthritis within 3 to 7 weeks after immunization. At the first appearance of clinical evidence of arthritis, mice were randomly divided into three groups, arthritic paws were injected periarticularly with either (1) AAV-OPG-eGFP at a titer of 10^8^ cfu/mL (*n* = 16), (2) AAV-LacZ at a titer of 10^8^ cfu/mL as viral controls (*n* = 16), or (3) virus-free medium as negative controls (*n* = 8).

Arthritic animals were clinically assessed 5 times a week and paw measurements were recorded 3 times a week for 8 weeks. The severity of arthritis in the affected paw was graded according to an established score system [25, 26] as follows: 0 = normal joint; 1 = mild/moderate visible edema and erythema; 2 = severe edema with distortion of paw and joint; and 3 = deformed paw or joint with ankylosis. The maximum score per mouse is 12 (4 paws) representing overall disease severity and progression in the animal.

All animals were sacrificed at week 8 after disease onset (except the group of mice for detection of transgene expression described in next paragraph). The arthritic paws were harvested and divided into 2 parts along the median longitudinal axis. One part was fixed in 10% formalin for histology process, while the remainder was stored at −80°C for molecular analyses.

### 2.4. Detection of Transgene Expression in Mouse Paw Tissues

To evaluate the transgene expression levels, two mice at each time were sacrificed at day 3, 7, 14, and 21 after in vivo AAV-OPG or AAV-LacZ vector transductions. Enzyme-linked immunosorbent assays (ELISA) were conducted on the supernatants of the paw tissues homogenates to examine OPG-transgene production. All antibodies for human OPG were obtained from R&D Systems (Minneapolis, MN). Tests were performed using the standardized protocol previously described [27, 28], and the protein levels were estimated against the standard curve corun on the same ELISA plate.

### 2.5. Histological Analysis

Paws were decalcified, paraffin-embedded, and sectioned to 6 *μ*m orientated longitudinally. The sections were routinely stained with hematoxylin and eosin (H & E). Toluidine blue staining was also performed for cartilage damage evaluation. Both H&E and Toluidine blue stained sections were examined to assess the severity of the arthritis using an established histological grading system [29]:

1. normal synovial membrane (2-3 cells thick) without cartilage changes,
2. inflammatory synovitis only,
3. pannus formation and superficial cartilage erosions,
4. major erosion of the cartilage and subchondral bone,
5. architectural changes and destructions.

### 2.6. MicroCT Analysis for Bone Mineral Density

An eXplore Locus Micro CT system (GE Medical Systems, London, ON, Canada) was used to evaluate bone mineral density changes during the experimental period. Mice were anesthetized (10 mg/kg of Xylazine and 120 mg/kg of Ketamine) and restrained during scans. The parameters for the scans were 45 *μ*m isotropic voxel size, 400 projections, 400 ms exposure time, 80 KW voltages, and 450 *μ*A current. Following acquisition and reconstruction of the 4 paws of each mouse, the image data were analyzed using a GEHC MicroView software with Analysis+ Version 1.0 (GE Healthcare, London, ON Canada) to generate VOI (volume of the interest) on the 2, 3, and 4 metatarsal or metacarpal joints (3 mm proximal and distal of the joint surface) and to calculate the bone mineral densities (BMD) of the subchondral bone within the VOI. The threshold was kept constant for all samples to eliminate the noise from the soft tissue.

### 2.7. Assessment of Gene Expression Profiles by RT-PCR

Real-time reverse transcriptase-polymerase chain reaction (RT-PCR) was performed to assess the influence of the gene transfer to the local mouse paw tissue. Gene expression of OPG, LacZ in the paw tissues were determined, and changes in expression of some proinflammatory cytokines and mediators including IL-1, TNF, IL-6, MMP3, and IFN-*γ* were also examined. Joints were harvested from arthritic mice postmortem at 56 days after arthritic onset. Total RNA from paw tissue homogenates were extracted following the manufacturer's instructions (Tel-Test, Friendswood, TX), and possible DNA contaminants were removed by RNase-free DNase (RQ1, Promega, Madison, WI, USA) treatment. The cDNA was reverse transcribed from 0.5 *μ*g of total RNA in a 20 *μ*L reaction mixture containing 1×PCR buffer, 500 *μ*M of each of deoxynucleotide triphosphates, 0.5 units/*μ*L of RNase inhibitor, 2.5 *μ*M random hexamers, 5.5 mM MgCl_2_, and 1.25 units/*μ*L of RT (Perkin-Elmer Cetus, Norwalk, CT). The reaction mixture was incubated in a Thermal Cycler (Perkin-Elmer Cetus) at 25°C for 10 minutes, 48°C for 25 minutes, followed by 95°C for 5 minutes. Real-time PCR was performed according to the manufacturer's instructions. To standardize target gene level with respect to variability in quality of RNA and cDNA, we used GAPDH, a housekeeping gene, as an internal control. Reaction mixtures of 25 *μ*L included 12.5 *μ*L of 2x SYBR Green PCR Master Mix (PE Biosystems, Foster City, CA), target gene primer pairs (at 400 nM final concentration), and 2 *μ*L cDNA. The primer pairs of the target genes used in this study (Table 1) were designed using a software Primer3 (http://frodo.wi.mit.edu/) with sequences from GenBank, NIH (http://www.ncbi.nlm.nih.gov/), and constructed by Invitrogen (Life Technologies, Grand Island, NY), with exception of primers for IL-1, TNF, and IL-6 which were purchased from Clontech Laboratories (Mountain View, CA). The reactions were run in MicroAmp optical 96-well reaction plates with MicroAmp optical caps for 40 cycles (95°C for 15 seconds, 60°C for 1 minute) in the ABI Prism 7700 Sequence Detector (Applied Biosystems) and the fluorescent signals were recorded dynamically. The values of threshold cycle (Ct) at which a statistically significant increase in reporter-dye signals is first detected, were recorded to calculate the relative quantification of target gene expression. With the Ct value of GAPDH as an internal control and mean Ct value of target gene expression from nonviral arthritic paw tissue as the calibrator samples (named 0% expression change), the comparative quantification of the target gene expressions of the samples from AAV-LacZ or AAV-OPG was calculated as described previously [30]. The reaction mixture after real-time PCR was electrophoresed on 1.8% agarose gels containing ethidium bromide to verify the correct amplification of the target gene.

### 2.8. Statistical Analysis

Power analysis (PS Power and Sample Size Calculations, http://biostat.mc.vanderbilt.edu/) was utilized to estimate that eight [8] mice per group were required for the study. Statistical analysis among groups was performed by one-way ANOVA test with the LSD formula for post hoc multiple comparisons (SPSS v16, Chicago, IL, USA). A *P*-value of 0.05 was considered as significant difference. Data are expressed as mean ± standard error of means.

## 3. Results

### 3.1. Transgene Production

**Figure 1:**
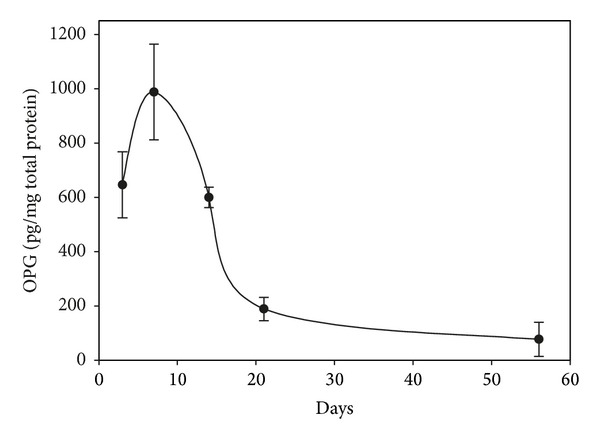
ELISA was performed to determine the transgene expression. hOPG protein was sustained expressed for at least 21 days, with the peak at 7 days following gene transfer.

ELISA was performed to determine the transgene expression. In virus-transduced arthritic joint homogenate, human OPG levels were maintained for at least 21 days, with the peak at 7 days following gene transfer (Figure 1). OPG protein level in blood serum was minimum, showing local effects of therapy. Mouse paws receiving AAV-lacZ vector injections revealed positive blue colored LacZ expressing cells under X-gal stains, similar to the reported previously [25]. There was no evidence of X-gal positive staining in uninjected paws or remote organs. 

### 3.2. Effects of OPG Gene Transfer on Clinical Progression of CIA

**Figure 2:**
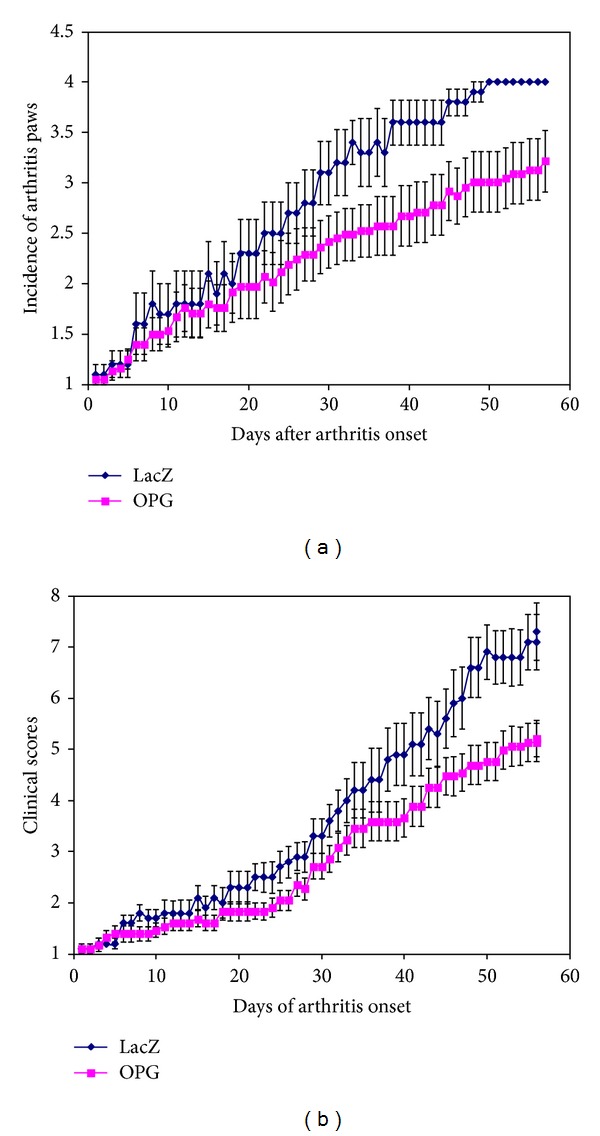
Summary of clinical assessment of CIA mice with or without OPG gene modification. Panel (a) shows the mean numbers of arthritic paws of each mouse during the disease progression, while panel (b) illustrates the arthritis scores of arthritic paws from the day of disease onset.

Therapeutic gene transfer of OPG significantly ameliorated the mean numbers of arthritic paws of each mouse during the disease progression (Figure 2(a)) and the mean arthritis scores of arthritic paws since the day that the first paw had arthritic changes (Figure 2(b)). The OPG gene transfer significantly diminished the severity of the disease progress as early as 3 weeks after onset. 

### 3.3. Histological Assessment of CIA following OPG Gene Modification

**Figure 3:**
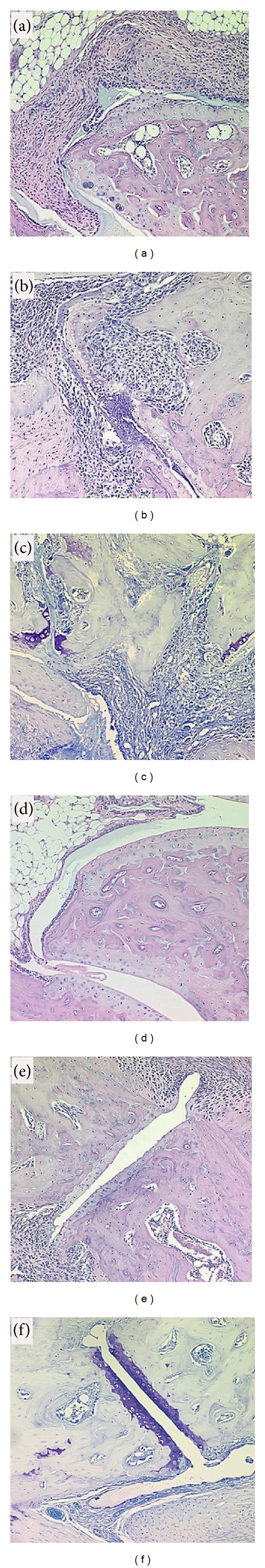
(a)–(c) Typical histological appearance of CIA mouse paws: (a) severe inflammatory cellular infiltration—synovitis and pannus formation; (b) bone and cartilage erosion; (c) toluidine blue stained section showing destruction of articular cartilage with loss of joint space and complete architecture distortion. Panels (d)–(f), example histological images of mouse paws after OPG gene modification: joint space was maintained and dramatically less bone and cartilage damage were obvious. (f) A Toluidine Blue stained paw section showing the protection effect on articulating cartilage.

**Figure 4:**
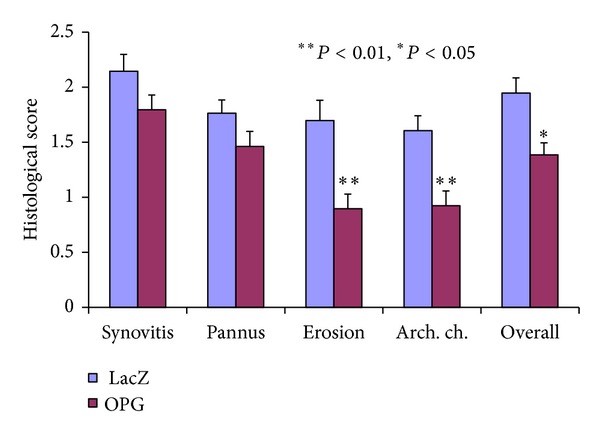
Summary of histological evaluation of arthritic paws with or without OPG gene transfer. Five categories are assessed on each paw section in a blinded fashion. OPG gene modification significantly ameliorate the disease progress, especially in bone/cartilage erosion and architecture disorganization.

Histological evaluation of the arthritic paws of negative control or LacZ-transduced mice revealed severe synovial inflammatory cellular infiltration and pannus formation, marked bone and cartilage erosion, and distortion of joint architecture (Figure 3). In vivo transduction of OPG dramatically protected against the cartilage and subchondral bone erosions (Figure 4, *P* < 0.05), although there were no significant ameliorations among the histological scores on synovitis and pannus formation, in comparison with the control animals. Toluidine staining showed that OPG gene transfer also effectively protected against the articular cartilage erosions and preserved the joint space and overall architectures (Figure 3). 

### 3.4. Bone Mineral Density Changes

**Figure 5:**
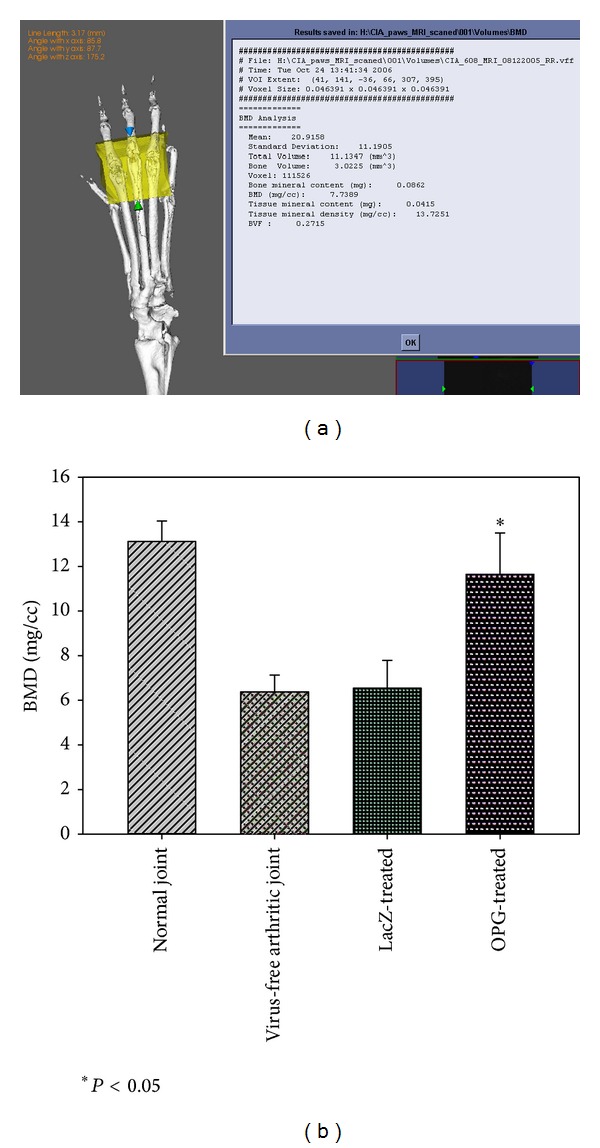
(a) GEHC MicroView software with Analysis + software to generate VOI on the 2, 3, and 4 metatarsal joints for BMD assessment. Panel (b) summarizes the bone mineral density readings among groups. OPG gene transfer significantly preserved the subchondral BMD compared to the untreated and LacZ-gene controls (*P* < 0.05).

MicroCT analysis was performed to quantify the subchondral bone mineral density (BMD) changes among groups of arthritic paws.  Figure 5(a) illustrates an example of the 3-D VOI on an arthritic paw CT image, and the analysis suggested that OPG gene transduction effectively protected against the subchondral bone degradation of the arthritic paws compared to the controls (Figure 5(b), *P < 0.05).

### 3.5. Molecular Analysis

**Figure 6:**
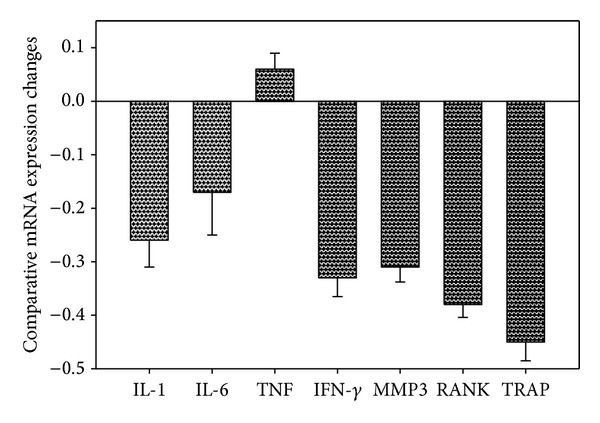
Gene expression profiles of the arthritic joint tissues following OPG gene transfer. The data are expressed as percentage changes of the comparative gene copies against readings from the LacZ-control gene transduced tissues. No mRNA expression change is expressed as 0.

Real-time PCR data suggested that there were significantly diminished mRNA expressions of TRAP, RANK, MMP3, and INF*γ* in the OPG-gene modified paw tissues, in comparison to the controls (Figure 6). While there was no significant influence of OPG gene transfer on TNF and IL-6 expression, in the local paw tissue, there was a reduction trend of IL-1 mRNA expression (*P* = 0.085). 

## 4. Discussion

Rheumatoid arthritis is an autoimmune disease that affects about 1% of the worldwide population. Bone destruction and loss are a serious clinical event in patients with rheumatoid arthritis (RA). Local joint destruction can cause joint instability and disability. Studies have shown the importance of CD4+ T cells, B cells, and cytokines such as RNAKL, TNF*α*, IL-1, and IL-17 in the pathogenesis of rheumatoid arthritis [3]. Cytokines exert direct effects on various types of cells in joint including chondrocytes, macrophages, and synovial fibroblasts, resulting in local inflammation and pannus formation, and activating different intracellular pathways to initiate osteoclast differentiation and localized joint destruction [3]. Immune cells and cytokines are critically responsible for the changes in bone turnover and bone mass that occur in rheumatoid arthritis. The balance of osteoclastogenic and antiosteoclastogenic mediators, thus, decides the fate of bone destruction.

RANKL and macrophage colony-stimulating factor (M-CSF) are critical factors in the differentiation and activation of osteoclasts [16]. Osteoclasts are the sole bone resorbing cells. RANKL plays a key role in bone resorption and it inserts its biological effects through binding to its receptor RANK on the surface of osteoclast precursors and mature osteoclasts, resulting in differentiation and activation of osteoclasts. Osteoprotegerin (OPG), a naturally present protein that binds RANKL without inserting downstream signal transduction [19], has been identified as a potent negative regulator of the osteoclastogenesis. Indeed, it is the balance between the expression of the stimulator of osteoclastogenesis RANKL and the inhibitor OPG that dictates the quantity of bone loss [31]. A recent study involving 64 RA patients with 60 healthy individuals indicated that there were significantly higher RANKL and lower OPG levels in sera from RA patients compared to those from healthy controls [32]. We hypothesized that locally elevating the OPG ratio over RANKL via viral vector-mediated OPG gene transfer would protect against and reverse the subchondral bone damages during the course of rheumatoid arthritis. Using the well-established collagen-induced arthritis model (CIA), this study clearly showed that over-expression of OPG significantly ameliorated the clinical course of CIA with relatively benign arthritis scores, and dramatically protected against subchondral bone loss and bone mineral density changes. Although OPG therapy alone appears to be not very effective in controlling the inflammatory natures of the disease such as synovitis and pannus formation, modification of OPG expression in the arthritis joint resulted in diminished gene expression of MMP3 and INF-*γ*. Since altered RANKL, OPG ratio blocked osteoclastogenesis in the arthritic paws (diminished TRAP and RANK expressions), the down-regulation of MMP3 and INF-*γ* may also due to reduction of the matured osteoclasts. This study also revealed that OPG gene transfer effectively protected against the articulating cartilage erosions. Diminishment of MMP3 and INF-*γ* gene expressions may be responsible for this therapeutic outcome. Further investigation is warranted for the underline molecular mechanism. 

The major problems in the treatment regimes for chronic inflammatory and destructive disorders such as rheumatoid arthritis include the lack of adequate suppressive agents and specific therapeutic delivery systems. Although still in infancy with many potential safety issues, therapeutic gene delivery system appears to have many advantages. Viral or nonviral vector-mediated gene transfer provides a rather efficient approach to deliver therapeutic gene(s) to the site of disease which may produce the therapeutic protein(s) in a persistent and localized manner. AAV vectors have shown some distinct advantages over other viral vectors such as infecting both dividing and non-dividing host cells and only provoking a very limited host immune response. In the current study, a single injection of AAV-OPG into the first diseased mouse paw at the onset day resulted in satisfactory therapeutic effects through the course of the experimentation. The transgene product OPG protein level at the transduced joint tissue could be detected for more than 3 weeks, and the improved clinical outcome was obvious, starting at 3 weeks after gene modification and lasting till the end of the experiment. In addition, there was no detected dissemination of the exogenous gene expression in mouse serum and remote organs. 

Overall, the current study suggests that the adeno-associated viral vector mediated in vivo gene transfer successfully transduced the transgene into arthritic joints which subsequently expressed OPG protein; the OPG gene transfer clearly ameliorated the disease progression in a mouse model of collagen-induced arthritis. Sustained OPG expression effectively inhibited the cartilage and subchondral bone erosions, preserved the architecture of arthritic joints, and maintained local bone mineral density. The therapeutic influence of OPG may be attributed to blockage of osteoclastogenesis and negative regulation of other arthritis-associated mediators such as IFN-*γ* and MMP3.

## Figures and Tables

**Table 1:** Primer sequences for the genes used in this study.

Target	Forward primer	Reverse primer
Human OPG	GGCAACACAGCTCACAAGAA	CTGGGTTTGCATGCCTTTAT
Mouse MMP3	GCCACTCCCTGGGACTCTACCAC	AGGGGATGCTGTGGGAGTTCCAT
Mouse IFN-*γ*	TGGAGGAACTGGCAAAAGGATG	CGCTTCCTGAGGCTGGATTC
Mouse RANK	GGGTGGGGCGCAGACTTCAC	ATGCCAGCAGCCTGCACCAG
Mouse TRAP	TCCTGGCTCAAAAAGCAGTT	ACATAGCCCACACCGTTCTC
